# Differential impact of comorbid depression and adjustment disorder on self-compassion in patients with anxiety disorders

**DOI:** 10.1192/j.eurpsy.2025.643

**Published:** 2025-08-26

**Authors:** K.-U. Lee, H.-S. Chun, S. Kim

**Affiliations:** 1 Psychiatry, Uijeongbu St. Mayr’s Hospital, College of Medicine, The Catholic University of Korea, Seoul, Korea, Republic Of

## Abstract

**Introduction:**

The capacity of self-compassion may contribute to the development or protection of psychiatric disorders.

**Objectives:**

This study aimed to examine the relationship between self-compassion and psychopathologies in anxiety disorders and to identify the impact of comorbid depression on self-compassion.

**Methods:**

Patients diagnosed with anxiety disorders were recruited from the outpatient clinic of the Catholic University of Korea. Psychiatric diagnoses were established through interviews based on DSM-5 criteria, conducted by an experienced psychiatrist. Demographic data were collected, and clinical status was evaluated using the Clinical Global Impression (CGI) scale. The severity of anxiety, depression, somatic symptoms, hypochondriasis, and self-compassion were assessed using the Generalized Anxiety Disorder-7 (GAD-7), Patient Health Questionnaire-9 (PHQ-9), Patient Health Questionnaire-15 (PHQ-15), Illness Attitude Scale (IAS), Cyberchondria Severity Scale (CSS-12), and Self-Compassion Scale (SCS), respectively.

**Results:**

The study included 121 participants with a mean age of 48.65 years (SD = 10.10), ranging from 20 to 70 years. The majority of the participants were female, comprising 91 (75.2%) of the sample, while 30 (24.8%) were male. A significant inverse relationship was observed between self-compassion (SCS total) and measures of general psychopathologies and hypochondriasis: PHQ-9 (r = -0.366, p < 0.001), GAD-7 (r = -0.348, p < 0.001), PHQ-15 (r = -0.349, p < 0.001), IAS (r = -0.293, p < 0.005), and CSS-12 (r = -0.208, p < 0.05). Also, strong negative correlations were found with all psychopathology measures, showing that higher negative self-compassion is significantly related to greater psychopathology: PHQ-9 (r = -0.479, p < 0.001), GAD-7 (r = -0.423, p < 0.001), PHQ-15 (r = -0.364, p < 0.001), IAS (r = -0.374, p < 0.001) and CSS-12 (r = -0.362, p < 0.001). An ANCOVA was conducted to assess group differences in self-compassion subscales among patients with anxiety disorders (N = 71), comorbid depressive and adjustment disorders (N = 22), and comorbid somatic symptom and related disorders (N = 25). The results showed a significant difference in the isolation subscale across the groups (F = 4.636, p = .012).

**Conclusions:**

The findings underscore the role of self-compassion in moderating psychopathology severity in anxiety disorders, suggesting that interventions targeting negative self-compassion may help mitigate emotional and physical symptoms in these patients

**Disclosure of Interest:**

None Declared

